# Role of Digital Health Technologies and Artificial Intelligence in Modern Public Health Surveillance

**DOI:** 10.7759/cureus.106690

**Published:** 2026-04-08

**Authors:** Rutuja Khobragade, Mohammed Kamran Shaikh, Harshal Gajanan Mendhe, Sonali K Borkar, Akshaya Gurdekar, Sahil Shendre, Seema Gupta

**Affiliations:** 1 Department of Community Medicine, School of Allied Health Sciences, Datta Meghe Institute of Higher Education and Research, Nagpur, IND; 2 Department of Community Medicine, Datta Meghe Medical College, Datta Meghe Institute of Higher Education and Research, Nagpur, IND; 3 Department of Orthodontics, Kothiwal Dental College and Research Centre, Moradabad, IND

**Keywords:** artificial intelligence in healthcare, digital, narrative review, public health, surveillance

## Abstract

Modern public health surveillance is undergoing a transformative shift, driven by digital health technologies and artificial intelligence (AI). Traditional surveillance systems, which rely heavily on manual reporting and delayed data aggregation, often struggle to provide the real-time insights necessary for timely interventions. The integration of digital tools, including electronic health records, mobile health applications, wearable devices, and Internet of Things (IoT) platforms, has enabled the continuous collection of large-scale data across diverse populations. These technologies facilitate the early detection of disease outbreaks, improve the monitoring of chronic conditions, and enhance population health management.

Artificial intelligence, including machine learning, natural language processing, and predictive analytics, further enhances the capabilities of digital surveillance systems by enabling automated data processing, pattern recognition, and forecasting. AI-driven models can analyze vast datasets from heterogeneous sources, including social media, environmental sensors, and clinical databases, to identify emerging health threats and predict disease trends. This capability has proven particularly valuable during global health crises, where rapid response and adaptive strategies are crucial. Artificial intelligence, including machine learning, natural language processing, and predictive analytics, further enhances the capabilities of digital surveillance systems by enabling automated data processing, pattern recognition, and forecasting. AI-driven models can analyze vast datasets from heterogeneous sources, including social media, environmental sensors, and clinical databases, to identify emerging health threats and predict disease trends. This capability has proven particularly valuable during global health crises, where rapid response and adaptive strategies are crucial.

Despite these advancements, several challenges persist, including concerns related to data privacy, ethical considerations, interoperability, and algorithmic bias. The digital divide also limits equitable access to these technologies, potentially exacerbating existing health inequities. Furthermore, the reliability and validity of AI models depend on the quality and representativeness of the data used for training the models.

This narrative review explores the evolving roles of digital health technologies and AI in modern public health surveillance. It examines key technological innovations, applications in disease monitoring and outbreak prediction, integration challenges, and ethical implications. By synthesizing the current evidence, this review highlights both the opportunities and limitations of these technologies and provides insights into future directions for building resilient, equitable, and data-driven public health surveillance systems.

## Introduction and background

The evolution of public health surveillance

Public health surveillance, the continuous, systematic collection, analysis, and interpretation of health data for action, has long been the cornerstone of disease prevention and control [1]. Historically, surveillance systems relied on passive reporting from clinicians, laboratory confirmation, and manual data aggregation, resulting in inevitable delays, underreporting, and fragmented situational awareness [2]. The HIV epidemic of the 1980s spurred the development of more structured surveillance, yet even at the turn of the century, most systems remained reactive, with lag times of days to weeks between disease occurrence and public health response [3,4]. The advent of digital technologies has begun to dismantle this traditional model, offering the possibility of real-time, granular, and predictive surveillance. From electronic health records (EHRs) and internet search queries to wearable sensors and genomic sequencing platforms, the sources of health data have proliferated exponentially, demanding a fundamental rethinking of how we detect, monitor, and respond to health threats [5,6].

Defining the paradigm shift

This transformation is driven by two interrelated domains: digital health technologies (DHTs) and artificial intelligence (AI). DHTs encompass a broad array of tools, including mobile health applications, wearable devices (e.g., smartwatches, continuous glucose monitors), social media platforms, wastewater surveillance sensors, and the digitization of clinical records [7]. These technologies generate high-velocity, high-volume data that, when harnessed appropriately, can capture population health signals far earlier than traditional indicators [8]. AI, particularly machine learning (ML), natural language processing (NLP), and computer vision, provides the analytical engine to process this data. NLP mines unstructured text from EHRs or social media for syndromic clues; ML models detect anomalies in disease incidence or mobility patterns; computer vision enables automated analysis of medical imaging for outbreak hotspots [9]. The convergence of DHTs and AI has catalyzed a paradigm shift: surveillance is no longer confined to designated public health institutions but is increasingly distributed across digital platforms, private sector databases, and citizen‑generated data streams [10]. This shift promises earlier outbreak detection, more precise targeting of interventions, and the ability to monitor noncommunicable diseases and social determinants in near real time.

Rationale for review

Despite the rapid proliferation of research and pilot implementations at the intersection of DHTs, AI, and surveillance, the evidence base remains fragmented. Studies appear across disparate disciplines, computer science, engineering, clinical medicine, epidemiology, and ethics, with little cross synthesis. Moreover, the field is characterized by heterogeneous methodologies, varying stages of implementation (from proof‑of‑concept to national deployment), and a lack of consensus on evaluation frameworks [11-13]. A scoping review is therefore the appropriate methodological approach. Unlike a systematic review that seeks to answer a narrow question with a critical appraisal of effect sizes, a scoping review aims to map the existing literature, identify key concepts, types of evidence, and gaps in research. This review will chart the breadth of DHTs and AI techniques applied to public health surveillance, delineate the domains in which they are most used, synthesize reported outcomes and implementation barriers, and highlight the ethical, legal, and social implications that must guide responsible innovation.

## Review

Search methodology

**Figure 1:**
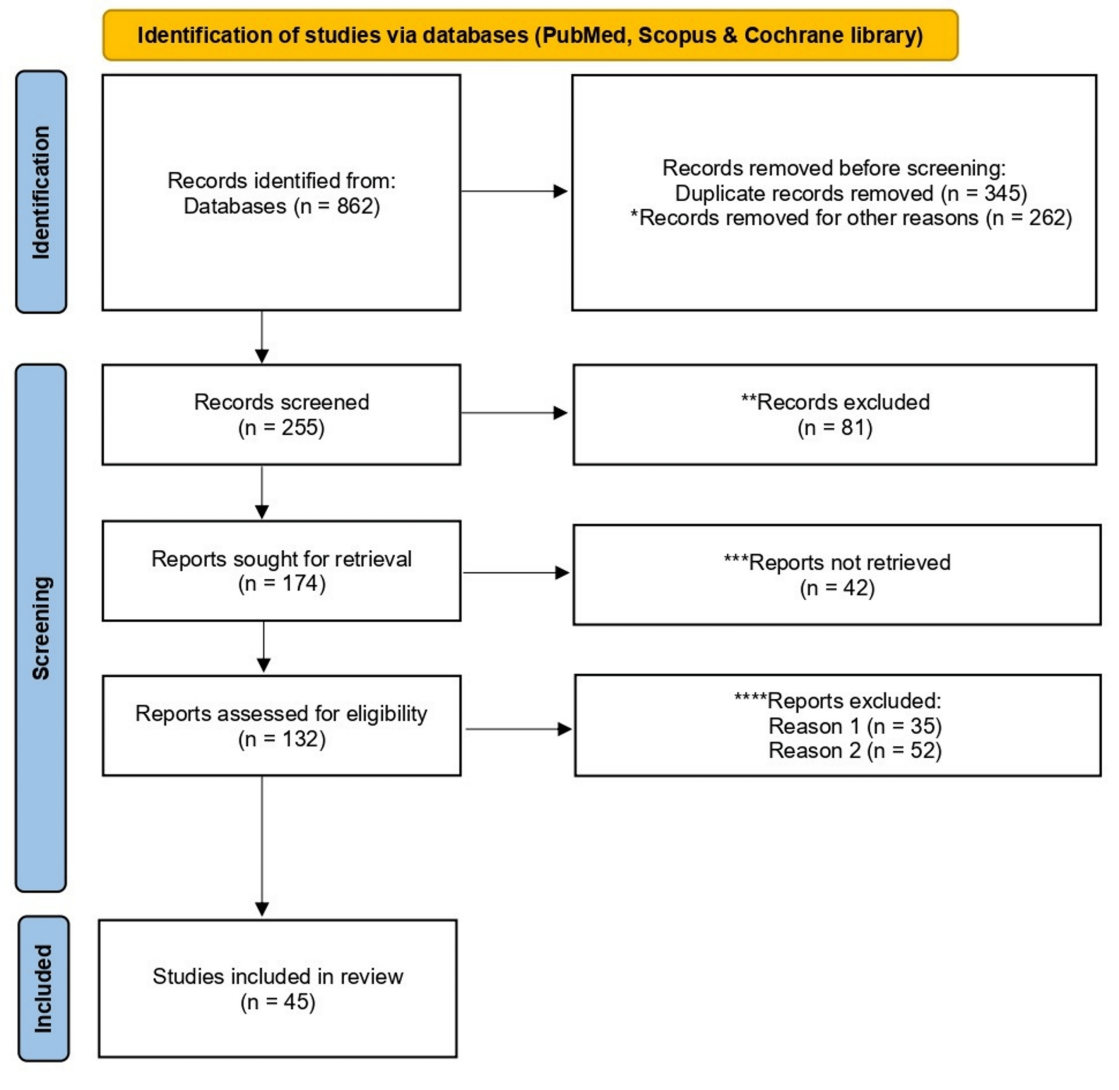
PRISMA 2020 flow diagram for reviews, which included searches of databases only. *Other reasons: non-English literature, editorials, wrong titles. **Excluded- Bio-surveillance not included, digital resources absent. ***Not retrieved- Subscription material, author not responded. **** Reason 1: Non-empirical studies, full text not available, Reason 2: Do not address the research questions.

Although this is a narrative review, the literature search and selection process adhered to the Preferred Reporting Items for Systematic Reviews and Meta-Analyses (PRISMA) 2020 guidelines for transparency and reproducibility [14]. Searches were conducted between January and March 2026 in PubMed/MEDLINE, Scopus, Web of Science, and Cochrane Library literature sources. Key terms combined Boolean operators: (“public health surveillance” OR “disease surveillance” OR “epidemiological surveillance”) AND (“digital health” OR mHealth OR eHealth OR wearable OR “Internet of Things” OR telehealth OR “electronic health records”) AND (“artificial intelligence” OR AI OR “machine learning” OR “deep learning” OR NLP OR “natural language processing”). The limits included English-language peer-reviewed articles, reviews, and reports published between 2015 and 2026 to capture post-digital acceleration and pandemic-era evidence (Figure 1).

The initial identification yielded 862 records. After duplicate removal (n = 345 excluded), title/abstract screening retained 255 articles. Full-text eligibility assessment (guided by inclusion criteria: relevance to surveillance applications and empirical or conceptual focus on digital/AI integration; exclusion: non-health surveillance, purely clinical AI, and pre-2015 studies) resulted in 132 included publications. After final full-text screening, only 45 studies were retained in the review. PRISMA flow (identification → screening → eligibility → inclusion) ensured systematic coverage while allowing narrative flexibility for thematic synthesis. Quality was appraised informally via peer review status and methodological clarity. The narrative synthesis organized the findings thematically under the subheadings below.

Evolution of public health surveillance: from traditional to digital

Traditional surveillance systems, rooted in mandatory notifiable disease reporting and periodic surveys, excel in structured environments but falter with the volume, velocity, and variety of modern data. Delays in data aggregation and underreporting in underserved areas limit early action. Digital transformation integrates real-time streams from citizen-generated data (apps, social media), passive sensors (wearables, environmental monitors), and institutional records (electronic health records [EHRs], laboratories) [15,16]. This enables “digital epidemiology” or “infoveillance,” in which signals emerge before clinical confirmation. AI layers predictive power, shifting surveillance from descriptive to anticipatory [17]. The COVID-19 response exemplified this: hybrid systems combined traditional case reporting with digital contact tracing and AI forecasting, dramatically reducing response times [15]. This evolution enhances sensitivity, specificity, and timeliness while addressing equity through mobile-first design in low-resource contexts.

Getachew et al. [18] highlighted how the COVID-19 pandemic accelerated the global adoption of digital health technologies, transforming healthcare delivery and public health surveillance. The authors emphasized the expanded use of telemedicine, mobile health applications, and digital platforms for real-time data sharing, which enabled continuity of care while minimizing the infection risk. This study also underscores the role of digital tools in outbreak tracking, contact tracing, and health communication. Importantly, it discusses challenges such as digital inequity, data privacy concerns, and infrastructure limitations, particularly in low- and middle-income countries, while advocating for the sustainable integration of digital health into future healthcare systems.

Key digital health technologies in surveillance

Digital health technologies provide a foundational data infrastructure. mHealth apps and wearables collect continuous biometric (heart rate, activity, and sleep) and symptom data, enabling syndromic surveillance and early anomaly detection. For example, smartphone-based fever tracking and GPS-enabled contact-tracing applications facilitated rapid isolation during outbreaks. EHRs and big data platforms aggregate clinical, demographic, and laboratory information for population-level trend analyses, with cloud integration supporting scalability [19]. IoT sensor networks, deployed in wastewater, air quality monitors, or smart cities, detect environmental precursors to vector-borne or respiratory diseases, providing real-time alerts [20]. Telehealth platforms extend surveillance to remote or quarantined populations via virtual triage and remote monitoring, conserving resources and minimizing exposure. Collectively, these create multimodal data ecosystems that AI can process for holistic insights, far surpassing the siloed traditional methods.

Keasberry et al. [21] provided a comprehensive overview of the clinical and organizational impacts of eHealth technologies in hospital practice. This study demonstrated that digital health systems, including electronic health records and telehealth platforms, improve care coordination, enhance clinical decision-making, and increase operational efficiency. Importantly, the authors highlighted that successful implementation depends on factors such as system interoperability, user training, and organizational readiness. These insights remain highly relevant for public health surveillance, where integrated digital infrastructure is essential for timely data sharing and effective response strategies.

Shakeri Hossein Abad et al. [22] conducted a systematic scoping review highlighting the growing role of digital technologies in public health surveillance. This study demonstrated that digital surveillance systems leverage diverse data sources, including social media, mobile applications, web-based platforms, and electronic health records, to enhance the timeliness and sensitivity of disease detection. The authors emphasized that such systems are particularly valuable for early outbreak identification and real-time monitoring, often complementing traditional surveillance methods. However, the review also underscored challenges related to data quality, privacy, standardization, and integration, indicating the need for robust governance frameworks to ensure the effective and ethical implementation of digital public health surveillance systems.

Artificial intelligence applications in public health surveillance

Staccini and Lau [23] highlighted the emerging role of artificial intelligence (AI) in enhancing precision public health through the analysis of social media data. Their work emphasizes that AI-driven techniques, particularly natural language processing (NLP), enable the early identification of health concerns by detecting patterns, sentiments, and signals in user-generated content. This approach facilitates faster recognition of outbreaks and evolving population health trends compared with conventional surveillance systems, thereby improving the timeliness and responsiveness. The authors also stressed the importance of integrating such digital signals with traditional epidemiological systems while addressing concerns related to data reliability, privacy, and ethical governance.

AI transforms raw digital data into actionable information [17,24]. Machine learning (ML) and deep learning (DL) models have demonstrated substantial utility in predictive analytics. Time-series forecasting using long short-term memory (LSTM) and recurrent neural networks (RNNs) has been widely applied to predict influenza and coronavirus disease 2019 (COVID-19) incidence by integrating climate variables, mobility patterns, and epidemiological data, often outperforming traditional statistical models in terms of predictive accuracy [25]. In addition, hybrid and ensemble models that combine ML with mechanistic epidemiological models are increasingly being used to improve the robustness and interpretability of forecasts [26,27].

Geospatial AI has further enhanced surveillance capabilities by enabling fine-scale mapping of disease risk. Techniques, such as random forests, gradient boosting, and convolutional neural networks (CNNs), integrate satellite imagery, land-use patterns, and climatic variables to identify hotspots for vector-borne diseases, such as dengue and malaria [27]. These approaches support targeted interventions by enabling location-specific risk stratification and resource allocation.

Natural language processing (NLP) and large language models (LLMs) have significantly expanded the scope of surveillance by enabling the extraction of insights from unstructured data sources, including social media platforms, online search queries, news reports, and electronic health record (EHR) narratives. This domain, often referred to as infodemiology, allows for the early detection of disease signals, misinformation trends, and public sentiment shifts, thereby complementing traditional surveillance systems [28]. AI-driven systems, such as digital epidemic intelligence platforms, can detect outbreaks days to weeks earlier than conventional reporting mechanisms [29].

Computer vision techniques further contribute to surveillance through the automated analysis of medical imaging, such as chest radiographs and computed tomography scans, for rapid disease detection and confirmation of outbreaks. In addition, video analytics and thermal imaging have been explored for mass screening in public spaces during pandemics. Generative AI models are emerging as powerful tools for simulating intervention scenarios, modeling disease spread under varying policy conditions, and supporting decision-making in uncertain environments [30,31].

Federated learning represents a critical advancement in privacy-preserving AI, enabling model training across decentralized datasets without requiring centralized data-sharing. This approach is particularly relevant in healthcare settings, where data sensitivity and regulatory constraints limit data aggregation. By enabling collaborative learning across institutions while maintaining data confidentiality, federated learning enhances the scalability and generalizability of surveillance models [32].

Recent advancements also include the integration of AI with Internet of Things (IoT)-enabled wearable devices and biosensors, facilitating continuous and real-time health monitoring. Supervised ML models, often combined with deep learning architectures, such as CNN-bidirectional LSTM (BiLSTM) networks with attention mechanisms, have achieved high accuracy in disease prediction and human activity recognition, with reported F1-scores exceeding 95% in certain applications [33,34]. Advanced signal-processing models, such as pulse photoplethysmography (Pulse-PPG), enable the precise extraction of physiological parameters, such as heart rate, in real-world ambulatory settings, outperforming traditional clinical baselines [35].

Beyond individual-level diagnostics, AI-driven surveillance systems play a crucial role in healthcare system optimization, including hospital resource management, workforce planning, and vaccine distribution strategies. These systems enable data-driven policymaking by integrating multi-source data streams and generating actionable insights for public health authorities to use. Furthermore, AI tools are increasingly being used to monitor and counter misinformation during health crises, thereby strengthening risk communication and public trust. [36]. Collectively, these advancements demonstrate that AI is transforming public health surveillance from a reactive system into a proactive, precision-driven framework capable of early detection, targeted interventions, and adaptive responses.

Case studies and real-world implementations

MacIntyre et al. [37] highlighted the critical role of artificial intelligence in strengthening epidemic intelligence through real-time monitoring, early warning systems, and automated outbreak alerts. This study emphasizes that AI-driven surveillance can enhance global preparedness by enabling the rapid detection of emerging infectious threats and supporting timely, data-driven public health responses. In China, hybrid IoT-EHR-AI platforms have enabled full-chain surveillance from screening to quarantine. For influenza, DL models on Japanese and US data achieved superior forecasting, and dengue prediction in Singapore reached 80% accuracy via random forests incorporating climate and mobility. Malaria and chickenpox surveillance in Korea benefited from the use of DNN/LSTM hybrids. NCD surveillance employs wearables and ML for risk stratification in chronic disease monitoring [18,20,38]. Low-resource examples include WhatsApp AI chatbots for symptom reporting and outbreak alerts in Africa. These cases demonstrate reduced morbidity through early interventions and optimized public health responses.

Challenges and limitations

Despite the significant promise of digital health technologies and AI in public health surveillance, several critical challenges hinder optimal implementation and scalability. One of the foremost concerns is data privacy and security, particularly in the context of integrating multi-source datasets, such as electronic health records, mobile applications, and social media platforms. The risk of data breaches and re-identification persists even in de-identified datasets, raising concerns under regulatory frameworks such as the General Data Protection Regulation (GDPR) and the Health Insurance Portability and Accountability Act (HIPAA). Ensuring secure data governance while maintaining usability remains a complex challenge in surveillance systems [39,40].

Another major limitation is algorithmic bias, which arises when AI models are trained on non-representative or skewed data sets. Such biases can lead to inaccurate predictions and exacerbate existing health disparities, particularly among marginalized and underserved populations. For example, biased training data may result in unequal disease risk prediction or misallocation of healthcare resources, undermining the principles of equity in public health [41]. Addressing this issue requires careful dataset curation, fairness-aware algorithms, and continuous validation across diverse populations.

Infrastructure and resource constraints further limit the widespread adoption of AI-driven surveillance, particularly in low- and middle-income countries. Reliable Internet connectivity, computational capacity, and access to digital devices are essential for implementing advanced surveillance systems. However, disparities in digital infrastructure and technical expertise create significant barriers, contributing to a global digital divide [42]. In addition, the lack of trained personnel capable of developing, deploying, and interpreting AI models hampers the effective utilization of these technologies.

The quality and reliability of the data used in AI models also present substantial challenges. Public health data are often incomplete, heterogeneous, and noisy, which can adversely affect the performance and generalizability of models. Inaccurate or biased input data may lead to flawed predictions, thereby reducing the trustworthiness of AI-based surveillance systems [43]. Moreover, overreliance on automated systems may introduce automation bias, wherein decision-makers place excessive trust in algorithmic outputs without critical evaluation.

Emerging concerns also include the environmental impact of AI technologies, particularly the high energy consumption associated with training large-scale deep learning models. This raises sustainability issues, especially when deploying AI solutions on a large scale in global health contexts [44]. Additionally, the digital divide continues to pose a major barrier to equitable implementation, as populations in resource-limited settings may lack access to digital tools, thereby limiting the inclusiveness of the surveillance systems.

To address these challenges, there is a growing emphasis on explainable artificial intelligence (XAI), which aims to improve the transparency and interpretability of AI models, thereby enhancing trust and accountability. Furthermore, robust regulatory frameworks, ethical guidelines, and continuous model validation are essential for ensuring responsible deployment. Capacity building through training programs, infrastructure development, and international collaboration is equally critical for bridging existing gaps and enabling equitable access to AI-driven public health surveillance systems [45].

Ethical, legal, and regulatory frameworks

Ethical deployment hinges on transparency, fairness, accountability, and human oversight (“human-in-the-loop”). The WHO principles emphasize human rights-aligned AI for health, whereas the OECD recommendations stress responsible innovation. The EU AI Act (2024) adopts a risk-based classification, prohibiting unacceptable uses and mandating high standards for high-risk surveillance systems. The European Health Data Space promotes secure data sharing. National policies must incorporate bias audits, consent mechanisms, and equity-impact assessments. Public health institutions should prioritize explainability, stakeholder engagement, and alignment with the public good to build trust and mitigate harm [46,47].

Future directions and recommendations

Emerging frontiers include AI-genomics integration for pathogen evolution tracking, blockchain technology for tamper-proof data sharing, and multimodal LLMs for holistic risk assessment. Hybrid human-AI systems, edge computing for real-time processing, and global federated platforms will enhance resilience. Policymakers should invest in an interoperable infrastructure, workforce training, and inclusive datasets. Public health agencies must develop AI governance frameworks, pilot equity-focused implementation, and foster cross-sector collaboration. Future research should prioritize longitudinal evaluations and cost-effectiveness studies. With responsible scaling, digital health and AI can create adaptive and equitable surveillance ecosystems capable of confronting future pandemics and chronic threats [48-50].

## Conclusions

Digital health technologies and AI have redefined the paradigm of public health surveillance, enabling a shift from reactive monitoring to proactive, data-driven decision-making. By integrating heterogeneous data sources, such as electronic health records, mobile platforms, environmental sensors, and social media, with advanced analytical techniques, these systems facilitate the early detection of outbreaks, accurate forecasting, and targeted interventions. This transformation enhances the efficiency, timeliness, and precision of public health responses, ultimately reducing morbidity and mortality.

However, the successful implementation of these innovations depends on addressing critical challenges, including data privacy and security, algorithmic bias, interoperability issues, and inequitable access to digital infrastructure. Strengthening ethical frameworks, regulatory policies, and global collaborations is essential to ensure responsible and inclusive deployment of AI in healthcare. Future efforts should focus on XAI, capacity building, and integration with traditional surveillance systems. Overall, digital health and AI hold immense potential for strengthening resilient, equitable, and sustainable public health surveillance systems worldwide.
